# CD44/CD24 immunophenotypes on clinicopathologic features of salivary glands malignant neoplasms

**DOI:** 10.1186/1746-1596-8-29

**Published:** 2013-02-18

**Authors:** Danilo Figueiredo Soave, João Paulo Oliveira da Costa, Giorgia Gobbi da Silveira, Renata Carolina Fraga Ianez, Lucinei Roberto de Oliveira, Silvia Vanessa Lourenço, Alfredo Ribeiro-Silva

**Affiliations:** 1 Department of Pathology, Ribeirão Preto Medical School, University of São Paulo, Avenida Bandeirantes; Number: 3900, Ribeirão Preto, São Paulo, 14049-900, Brazil; 2 Department of Pathology, A.C. Camargo Hospital, São Paulo, Brazil; 3 General Pathology Department, Dental School, University of São Paulo, São Paulo, Brazil

**Keywords:** Salivary gland, Salivary gland neoplasm, CD44, CD24, Clinicopathologic features

## Abstract

**Background:**

Salivary Glands Malignant Neoplasms (SGMNs) account for 3-6% of head and neck cancers and 0.3% of all cancers. Tumor cells that express CD44 and CD24 exhibit a stem-cell-like behavior. CD44 is the binding site for hyaluronic acid, and CD24 is a receptor that interacts with P-selectin to induce metastasis and tumor progression. The present study aims to evaluate the expression of CD44 and CD24 on SGMNs and correlated these data with several clinicopathologic features.

**Methods:**

Immunohistochemical stains for CD44 and CD24 were performed on tissue microarrays containing SGMN samples from 69 patients. The CD44, CD24 and CD44/CD24 expression phenotypes were correlated to patient clinicopathologic features and outcome.

**Results:**

CD44 expression was associated with the primary site of neoplasm (p = 0.046). CD24 was associated with clinical stage III/IV (p = 0.008), T stage (p = 0,27) and lymph node (p = 0,001). The CD44/CD24 profiles were associated with the primary site of injury (p = 0.005), lymph node (p = 0.011) and T stage (p = 0.023). Univariate analysis showed a significant relationship between clinical staging and disease- free survival (p = 0.009), and the overall survival presents relation with male gender (p = 0.011) and metastasis (p = 0.027).

**Conclusion:**

In summary, our investigation confirms that the clinical stage, in accordance with the literature, is the main prognostic factor for SGMN. Additionally, we have presented some evidence that the analysis of isolated CD44 and CD24 immunoexpression or the two combined markers could give prognostic information associated to clinicopathologic features in SGMN.

**Virtual Slides:**

The virtual slide(s) for this article can be found here: http://www.diagnosticpathology.diagnomx.eu/vs/1284611098470676.

## Background

Salivary Glands Malignant Neoplasms (SGMNs) are relatively rare malignant solid tumors. They represent a group of tumors that present favorable features concerning to loco-regional invasion and metastasis [1]. The estimated global incidence rate of SGMN varies from 0.4 to 2.6 cases per 100,000 people [2]. Because SGMNs account for 3% to 6% of all head and neck cancers, it represents only 0.3% of all human malignancies [3].

Following the discovery of hematopoietic stem cells, the “cancer stem cell” theory was proposed [4-7]. Currently, the most accepted theory for the etiopathogenesis of cancer is that the tumor starts as a single cell that shares some features with normal stem cells (SC) [4-6]. This cancer stem cell (CSC) may be involved in cancer initiation, maintenance of tumor growth, progression, tumor recurrence [6] and may also predict tumor treatment outcome [4]. SC and CSC have been identified in most tissues and solid tumors including in the brain [8], colon [9], breast [10] melanocytic cells [11], pancreas [12] and prostate [13].

There is evidence that tumor cells which express CD44 and CD24, exhibit a stem-cell-like behavior. CD44 is an important receptor for hyaluronate (HA) [14]. The functions of this transmembrane receptor include coordination of cell motility, cell-cell adhesion, lymphocyte activation [15], cell migration and cellular-extracellular matrix interaction [14]. Besides the interaction with HA, the CD44 protein has also been shown to interact with other proteins in the extracellular matrix including fibronectin, collagen types I and IV, serglycin and osteopontin [16]. The CD44 receptor is one of the most frequently studied SC markers [17], and CD44 expression is associated with epithelial tumors, although the results show different correlations in each tissue analyzed. Increased CD44 expression has been observed in aggressive gastric cancer [18]. Wielenga et al. [19] demonstrated that CD44 variant, v6, is strongly related to tumor progression, supporting the concept that CD44 plays an important role in human colorectal tumor metastasis. However, irregular or low expression of CD44 was related to poor prognoses in oral squamous cells carcinoma [16,20-22].

CD24 is a glycosylphosphatidylinositol-linked cell surface glycoprotein [23]. It has been identified as a P-selectin ligand and adhesion receptor for platelets and activated endothelial cells [24]. The CD24 protein is expressed in keratinocytes, renal tubules, regenerating muscles and the developing brain and pancreas [23]. It may be involve as a regulator factor for the control of cell proliferation, cell adhesion and apoptosis. However, the expression and physiological function of CD24 in human malignancies has not yet been completely elucidated [23]. In human carcinomas, there is evidence that CD24 expression is related to prognosis [25] and may contribute to metastasizing tumor cells [24], but the relationship between CD24 and prognosis is not completely understood.

Some studies have reported the correlation between CSC and unregulated expression of CD44 and CD24 [10,12]. However, the existence of CSCs in SGMN and their (CSCs) putative influence on the behavior of SGMN merit further investigation. Thus, the present study aim to investigates the expression of CD44 and CD24 in SGMNs and the association of CD44/CD24 immunophenotypes to the clinicopathologic features of SGMNs.

## Methods

### Subjects

Sixty-nine cases of SGMN, diagnosed between 1990 and 2009, were retrieved from the medical files of the Department of Pathology, Ribeirão Preto Medical School, University of São Paulo, Brazil. The medical files were analyzed to collect information on age, gender, primary anatomic localization, tumor evolution, history of smoking and alcohol intake, histological classification, nodal status, treatment, tumor recurrences, disease-free survival (DFS) and overall survival (OS). The study protocol conformed to the ethical guidelines of the 1975 Declaration of Helsinki and was carried out with the approval of the Ribeirão Preto Medical School Human Research Ethics Committee.

The following inclusion criteria were applied: 1) sufficient of clinicopathologic data; 2) histological diagnosis of mucoepidermoid carcinoma (MC), adenoid cystic carcinoma (ACC), acinic cell adenocarcinoma (ACA), adenocarcinoma (nos) (A (nos)), carcinoma ex-pleomorphic adenoma (Cex-PA), salivary duct carcinoma (SDC), polymorphous low-grade adenocarcinoma (PLGA), basal cell adenocarcinoma (BCA) or malignant myoepithelial neoplasm (MMN) (diagnoses as defined by the World Health Organization)) [26]; 3) no previous head and neck cancer; 4) no prior oncologic therapy. In accordance with Wittekind et al. [27] all cases were staged according to TMN classification.

For statistical analysis, the smoking and alcohol intake history was dichotomized: the patients were classified as “current consumers” or “never consumers”. Current consumers of tobacco were those individuals who used a cigarette, pipe, and/or cigar daily, and current alcohol consumption was defined as daily use of wine, beer, and/or other distilled drinks. “Never consumers” were those who had quit alcohol and/or tobacco consumption more than 12 months prior to diagnosis.

### Tissue Micro-Array (TMA)

Hematoxylin and eosin (H&E) stained slides from 69 cases of SGMNs were reviewed by an experienced pathologist (ARS) to detect the most significant tumor areas. These areas were then selected for assembly into the TMA paraffin block. Representative 2-mm cores from selected areas from each tumor were detached and allocated to a recipient paraffin block using a manual tissue-arraying instrument (“Tissue Micro-Array Builder,” Histopathology, Ltd., Akác u. 8, 7632 Pécs, Hungary, Baranya). Three-micrometer-thick sections were cut from the TMA paraffin blocks using a paraffin tape-transfer system (Instrumedics, Saint Louis, MO, USA). The first and final sections were stained with H&E to confirm the presence of SGMN by light microscopy.

### Immunohistochemistry (IHC)

All 69 samples were fixed in 4% neutral formalin and embedded in paraffin. Three-micrometer-thick sections were cut from the TMA paraffin blocks. The sections were dewaxed in xylene and rehydrated by immersing in a series of graded alcohols. Endogenous peroxidases were blocked using a solution containing 0.3% hydrogen peroxide for 30 min. The paraffin sections were then placed in 10 mM citrate buffer and submitted to heat retrieval, using a vapor lock, for 40 min. Following antigen retrieval, the slides were allowed to cool at room temperature for 30 min, and the sections were then incubated with the primary antibody at 4°C overnight. The dilution, clone and source of the antibodies used in this study were CD44 (mouse monoclonal antibody 1:100, clone DF1485, Novocastra, Newcastle upon Tyne, United Kingdom) and CD24 (mouse monoclonal antibody 1:50, clone SN3b, Thermo Scientific, Fremont, CA, USA). Following overnight incubation with the appropriate primary antibody, all sections were incubated with post-primary solution, for 30 minutes, and then incubated with the polymer (both provided by Biocare Medical Mach 4 Universal Polymer Detection System, Concord, California, USA) for 30 minutes. The reaction was developed by adding diaminobenzidine (DAB), and the sections were counterstained with hematoxylin. Finally, the slides were dehydrated in a graded series of alcohols and mounted with permount. The percentages of CD44 and CD24 positive cancer cells were determined by two observers (ARS and LRO) with a blind fashion. Discrepancies between the observers were resolved by conference, achieving consensus for each case.

The IHC reaction was carried out on two slides at different depth levels through the tumor to increase the accuracy and the robustness of the array-based data. Negative controls were prepared by omitting the primary antibody, for the both antibodies were used human tonsil tissue as positive controls. In accordance with protocols devised by Honeth and colleagues [28] CD44 staining was detect in the membrane and scoring was as follows: 0, 0% positive tumor cells; 1, 1% to 10% positive cells; 2, 11% to 50% positive cells; 3, 51% to 75% positive cells; and 4, 76% to 100% positive cells. The cases classified as 1 or superior were considered positive to quantitative analysis. The quantification of CD24 was evaluated in accordance with protocols devised by Kristiansen and colleagues [29]. CD24 staining was detected in the membrane and cytoplasm. Negative controls showed no signs of staining, as positive controls were used human tonsil tissue. Cases presenting unequivocal staining [29] were classified as positive.

**Figure 1:**
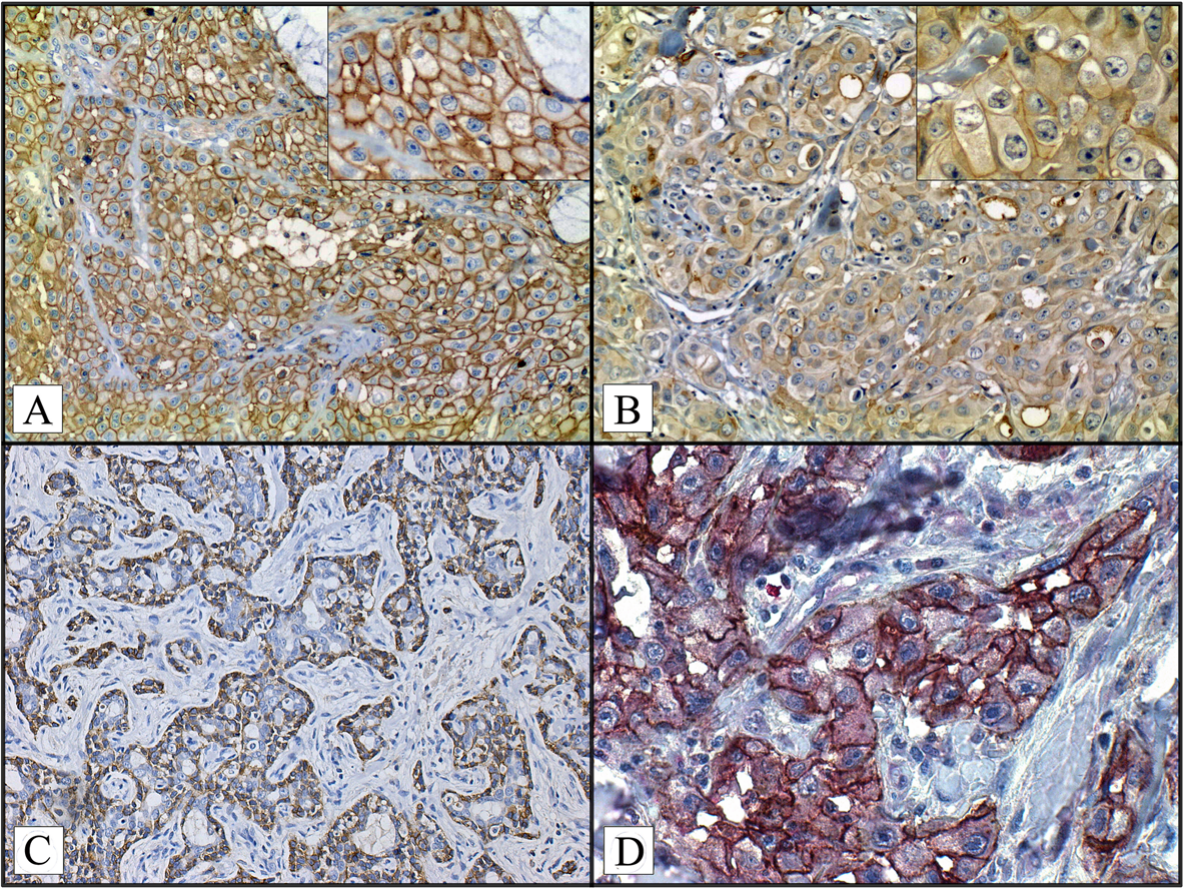
**Immunohistochemical staining for CD44 and CD24 A) CD44 positive (membrane staining) in case of mucoepidermoid carcinoma (× 200) ; B) CD24 positive (membrane and cytoplasmic staining) in case of in case of mucoepidermoid carcinoma.** (× 400) (detail in right upper panel) **C**) CD44 positive (membrane staining was observed only on myoepithelial component) in case of adenoid cystic carcinoma (× 200), **D**) Double immunohistochemical reaction of CD44 and CD24 in mucoepidermoid carcinoma. CD44 positive are indicated in brown (DAB, membrane staining). CD24 positive cell are indicated in red (Fast red, cytoplasm), magnification x400.

For prognostic investigation and survival analysis, the immunophenotypes patterns were grouped: CD44 + /CD24+ (CD44 positive case/CD24 positive CD44 + /CD24- (CD44 positive case/CD24 negative case), CD44-/CD24+ (CD44 negative case/CD24 positive case), CD44-/CD24- (CD44 negative case/CD24 negative case). The double-immunolabeling study was performed according to the manufacturer’s protocol (EnVision Doublestain System; Dako, Carpinteria, CA, USA). The CD44 was developed with DAB (brown), and the CD24 was developed with fast red (red) (Figure 1D).

### Statistical analysis

Statistical analyses were performed using the commercially available PASW Statistics 18.0 software (Chicago, IL, USA). The correlations of CD44, CD24 expression and clinicopathologic features were tested with contingency tables using Fisher’s exact test (two variables) or χ2 (three or more variables) tests. The OS rate was defined as the interval between the diagnosis and the date of death (uncensored data) or the date of the last available clinical information (censored data). The DFS was defined as the time from diagnosis until the occurrence of local recurrence or metastasis. Univariate analyses were performed using Kaplan-Meier curves and the log-rank test to compare and estimate cumulative survival rates. Multivariate Cox proportional hazard regression was used to correlate the risk factors with independent prognostic properties. Due to their limited number, the CD44-/CD24+ cases were removed from analyses. All tests were two- tailed and P-values < 0.05 were considered statistical significance.

## Results

### Patient demographics and clinical data

A total of 69 patients with SGMN (28 men (40.5%) and 41 women (59.5%) were used for the immunohistochemical study. Age ranged from 13 to 85 years with a mean age of 54,8 years. The age of male range from 23 and 85 years, with a mean age of 58 years. The ages of female were range from 13 and 85 years (average 51.5 years). Information on history of smoking and alcohol intake showed a total of 23 (33.3%) tobacco users and 14 (20.3%) alcohol users.

In the present study, 27 (39.1%) tumors were found in the parotid gland, 13 (18.8%) tumors in the submandibular gland, 27 (39.1%) tumors in the minor salivary glands and 2 tumors (2.9%) in the sublingual gland.

The neoplasms were classified as mucoepidermoid carcinoma (11 cases (15.9%)), adenoid cystic carcinoma (34 cases (49.3%)), acinic cell adenocarcinoma (4 cases (5.8%)), adenocarcinoma ,NOS (5 cases (7.2%)), polymorphous low-grade adenocarcinoma (3 cases (4.3%)), basal cell adenocarcinoma (5 cases (7.2%)), carcinoma ex- pleomorphic adenoma (4 cases (5.8%)), and salivary duct carcinoma (3 cases (4.3%)).

The diameter of the tumors ranged from 0.3 cm to 8.0 cm. Twenty-five cases (36.2%) were classified as T1 tumors and 17 cases (24.6%) were T2 tumors, 16 cases (23.2%) were T3 tumors, 11 cases (15.9%) were T4 tumors. In these samples, 16 cases (23.2%) were classified as being in stage I, 8 cases (11.6%) were in stage II, 11 (15.9%) cases were in stage III and 34 cases (49.3%) were classified as being in stage IV. All 69 patients underwent surgical treatment. Following the surgical treatment, 50 patients received adjuvant therapy, 30 patients received only radiotherapy, 4 received only chemotherapy and 16 patients were received both chemotherapy and radiotherapy.

Metastases developed in 26 patients, 24 patients developed loco-regional recurrence, 11 patients developed lymph node metastasis and 21 patients died during the course of the study. The disease-free survival and the overall survival ranged from 1 month to 248 months (the DFS mean was 52 months and OS mean was 62 months). The clinical features are summarized in Table 1.

**Table 1:** Clinical features of the 69 SGMN patients

		**Male**		**Female**		**Total**	
			**%**		**%**		**%**
Gender		28	40.6	41	59.4	69	100.00
Age	≤ 60	19	27.5	22	31.9	41	59.3
	> 60	9	13.0	19	27.5	28	40.7
Smoking intake	Yes	14	20.3	9	13.0	23	33.3
	No	14	20.3	32	46.4	46	66.7
Alcohol intake	Yes	9	13.0	5	7.2	14	20.3
	No	19	27.5	36	52.2	55	70.3
Disease Classification	MC	7	10.1	4	5.8	11	15.9
	ACC	11	15.9	23	33.3	34	49.3
	ACA	1	1.4	3	4.3	4	5.8
	A.(nos)	4	5.8	1	1.4	5	7.2
	BCA	2	2.9	3	4.3	5	7.2
	PLGA	2	2.9	1	1.4	3	4.3
	Cex-PA	0	0.0	4	5.8	4	5.8
	SDC	1	1.4	2	2.9	3	4.3
PLS^a^	PG^b^	10	14.5	17	24.6	26	37.7
	SubM.G^c^	8	11.6	5	7.2	13	18.8
	SubL.G^d^	0	0.0	2	2.9	2	2.9
	MinorG^e^	10	14.5	17	24.6	27	39.1
Tumor Size	T1	5	7.2	20	30.0	25	36.2
	T2	12	17.4	5	7.2	17	24.6
	T3	5	7.2	11	15.9	16	23.2
	T4	6	8.7	5	7.2	11	15.9
Clinical Stage	I	2	2.9	14	20.3	16	23.2
	II	7	10.1	1	1.4	8	11.6
	III	2	2.9	9	13.0	11	15.9
	VI	17	24.6	17	24.6	34	49.3
Treatment	Surgery	5	7.2	14	20.3	19	27.5
	S. RT	11	15.9	19	27.5	30	43.5
	S. CT.	3	4.3	1	1.4	4	5.8
	S. RT and CT	9	13.0	7	10.1	16	23.2
Lymph node	Negative	24	34.8	34	49.3	58	84.1
	Positive	4	5.8	7	10.1	11	15.9
Metastasis	No	15	21.7	28	40.6	43	62.3
	Yes	13	18.8	13	18.8	26	37.7
Loco-regional	No	17	24.6	28	40.6	45	65.2
recurrence	Yes	11	15.9	13	18.8	24	34.8
Death	No	15	21.7	33	47.8	48	69.6
	Yes	13	18.8	8	11.6	21	30.4

RT: Radiotherapy; CT: Chemotherapy; ^a^ PLS: Primary Lesion Site; ^b^ PG: Parotid Gland; ^c^ SubM.G: Submandibular Gland; ^d^ SubL.G: Sublingual Gland; ^e^ MinorG: Minor Salivary Glands. MC: mucoepidermoid carcinoma; ACC: adenoid cystic carcinoma; ACA: acinic cell adenocarcinoma; A (nos): adenocarcinoma; Cex-PA: carcinoma ex-pleomorphic adenoma; SDC: salivary duct carcinoma; PLGA: polymorphous low-grade adenocarcinoma; BCA: basal cell adenocarcinoma (BCA); MMN: malignant myoepithelial neoplasm.

### Statistical analysis of SGMN clinicopathologic features

Clinicopathologic features (gender, age, smoking and alcohol intake history, lymph node metastases, clinical and T stage, loco-regional recurrence, metastasis and death) were tested with contingency tables using Fisher’s exact test (two variables) or χ2 (three or more variables) tests and P-values < 0.05 were considered statistical significance. The present data shown association between gender and death, were observed that male patients had a high number of death events during the course of the study (p = 0.017), The metastasis process has also presents a relation with death (p = 0,007), positive metastasis patients presents increased death proportion. Our research has also found association between local-recurrence, clinical stage and metastases. The analysis demonstrates a higher proportion of patients positives to local-recurrence associated to advanced clinical stage (III and IV) (p = 0.044) and were observed, during the course of the study, patients presenting local-recurrence had a high propensity to distant metastasis (p = 0.010). All the remaining statistical comparison of clinicopathologic features were considered no statistical significant.

### Immunohistochemical findings and their relationship to clinicopathologic features

The positive controls to CD44 and CD24 were used human tonsil tissue. We observed in normal salivary glands CD44 widely expressed by acinar cells (membranous stain), however CD24 do not present staining (membranous and citoplasmatic stain), in normal gland. CD44 membrane expression (Figure 1A) was verified in 50 of 69 cases (72.4%). Eighteen cases were positive for CD24 (Figure 1B).

The CD44 expression on Adenoid cystic (26 cases) was moderate to strong staining, cytoplasmic and membranous was observed on myoepithelial component. Mucoepidermoid carcinoma positive cases (7 cases) showed weak to strong patchy membranous epidermoid cell staining. Acinic cell carcinoma (4 cases) and Carcinoma Ex-pleomorfic Adenoma (3 cases) positive cases displayed focal weak to moderate membranous staining. The Polymorphous low-grade (1 case) showed moderate patchy of membranous staining, Adenocarcinoma (NOS) 5 cases and salivary duct carcinoma (2 cases) exhibited moderate focal areas of membranous staining, and Basal Cell Adenocarcinoma (2 cases) exhibited weak diffuse membranous staining throughout the tumoral islet. However, the CD24 displayed focal weak to moderate membranous/citoplasmatic staining in SGMN (mucoepidermoid carcinoma 3 cases), adenoid cystic carcinoma (12 cases), carcinoma ex-pleomorphic adenoma (1 case), and salivary duct carcinoma (2 cases).

The CD44 expression was associated with major salivary gland topography (p = 0.046). CD24 expression was associated with T stage (p = 0,27) and lymphnode metastasis (p = 0,001). The 69 cases were grouped according to CD44/CD24 profiles as follows: 36 cases (52.2%) were CD44+/CD24-; 15 cases (21.7%); were CD44-/CD24-; 14 cases (20.3%) were CD44+/CD24+ (Figure 1D) and 4 cases (5.8%) were CD44-/CD24+. The profiles CD44/CD24 correlated with primary lesion site (p = 0,005), lymphnode metastasis (p = 0,011) and T stage (p = 0,023). All of the immunohistochemical findings and relationships to clinicopathologic features concerning the CD44/CD24 profiles are summarized in Tables 2 and 3.

**Table 2:** Association of CD44 and CD24 with clinicopathological variables in 69 SGMN patients

		**CD44**			**CD24**		
		**+**	**-**	**p**	**+**	**-**	**p**
Gender	Male	19 (13.0%)	9 (13.0%)	0.330	6 (8.7%)	22 (31.9%)	0.330
	Female	31 (44.9%)	10 (14.5%)		12 (17.4%)	29 (42.0%)	
Age	≤ 60	30 (43.5%)	11 (15.9%)	0.543	10 (14.5%)	31 (44.9%)	0.453
	> 60	20 (29.0%)	8 (11.6%)		8 (11.6%)	20 (29.0%)	
Smoking intake	Yes	15 (21.7%)	8 (11.6%)	0.250	5 (7.2%)	18 (26.1%)	0.392
	No	35 (50.7%)	11 (15.9%)		13 (18.8%)	33 (47.8%)	
Alcohol intake	Yes	9 (13.0%)	5 (7.2%)	0.324	5 (7.2%)	9 (13.0%)	0.275
	No	41 (59.4%)	14 (20.3%)		13 (18.8%)	42 (60.9%)	
PLS^a^	Major^b^	34 (49.3%)	8 (11.6%)	0.046	11 (15.9%)	31 (44.9%)	0.605
	Minor^c^	16 (23.2%)	11 (15.9%)		7 (10.1%)	20 (29.0%)	
Tumor size	T1/T2	28 (40.6%)	14 (20.3%)	0.142	7 (10.1%)	35 (50.7%)	0.027
	T3/T4	22 (31.9%)	5 (7.2%)		11 (15.9%)	16 (23.2%)	
Clinical Stage	I / II	19 (27.5%)	6 (8.7%)	0.419	2 (2.9%)	23 (33.3%)	0.008
	III / VI	31 (44.9%)	13 (18.8%)		16 (23.2%)	28 (40.6%)	
Treatment	Surgery	14 (20.3%)	5 (7.2%)	0.572	4 (5.8%)	15 (21.7%)	0.398
	Sur. Adj. T.^d^	36 (52.2%)	14 (20.3%)		14 (20.3%)	36 (52.2%)	
Metastasis	No	32 (46.4%)	11 (15.9%)	0.421	10 (14.5%)	33 (47.8%)	0.339
	Yes	18 (26.1%)	8 (11.6%)		8 (11.6%)	18 (26.1%)	
loco-regional	No	33 (47.8%)	12 (17.4%)	0.519	10 (14.5%)	35 (50.7%)	0.236
recurrence	Yes	17 (24.6%)	7 (10.1%)		8 (11.6%)	16 (23.2%)	
Lymph node	Negative	43 (62.3%)	15 (21.7%)	0.352	10 (14.5%)	48 (69.6%)	0.001
	Positive	7 (10.1%)	4 (5.8%)		8 (11.6%)	3 (4.3%)	
Death	No	36 (52.2%)	12 (17.4%)	0.332	14 (20.3%)	34 (49.3%)	0.285
	Yes	14 (20.3%)	7 (10.1%)		4 (5.8%)	17 (24.6%)	
CD24	Negative	36 (52.2%)	15 (21.7%)				0.398
	Positive	14 (20.3%)	4 (5.8%)				
CD44	Negative				4 (5.8%)	15 (21.7%)	0.398
	Positive				14 (20.3%)	36 (52.2%)	

^a^**PLS:** Primary Lesion Site; ^b^**Major:** Major Salivary Glands **(**Parotid Gland/Submandibular Gland/Sublingual Gland); ^c^**Minor;** Minor Salivary Glands; ^**d**^**Sur. Adj. T.:** Surgery with adjuvant therapy. Statistically significant difference (<0.05) by Fisher’s exact test (two variables) or χ2 (three or more variables) tests.

**Table 3:** Association of CD44/CD24 immunophenotypes with clinicopathological variables in 69 SGMN patients

		**CD44/CD24 profile**^**a**^					
		**+/+**	**+/−**	**−/+**	**−/−**	**Total**	**p**
Gender	Male	2 (2.9%)	17 (24.6%)	4 (5.8%)	5 (7.2%)	28 (40.6%)	0.091
	Female	12 (17.4%)	19 (27.5%)	0 (0.0%)	10 (14.5%)	41 (59.4%)	
Age	≤ 60	8 (11.6%)	22 (31.9%)	2 (2.9%)	9 (13.0%)	41 (59.4%)	0.967
	> 60	6 (8.7%)	14 (20.3%)	2 (2.9%)	6 (8.7%)	28 (40.6%)	
Smoking intake	Yes	3 (4.3%)	12 (17.4%)	2 (2.9%)	6 (8.7%)	23 (33.3%)	0.554
	No	11 (15.9%)	24 (34.8%)	2 (2.9%)	9 (13.0%)	46 (66.7%)	
Alcohol intake	Yes	2 (2.9%)	7 (10.1%)	3 (4.3%)	2 (2.9%)	14 (20.3%)	0.831
	No	12 (17.4%)	29 (42.0%)	1 (1.4%)	14 (21.7%)	55 (79.7%)	
PLS^b^	Major^c^	7 (10.1%)	27 (39.1%)	4 (5.8%)	4 (5.8%)	42 (60.9%)	0,005
	Minor^d^	7 (10.1%)	9 (13.0%)	0 (0.0%)	11 (15.9%)	27 (39.1%)	
Tumor size	T1/T2	4 (5.8%)	24 (34.8%)	3 (4.3%)	11 (15.9%)	42 (60.8%)	0.023
	T3/T4	10 (14.5%)	12 (17.4%)	1 (1.4%)	4 (5.8%)	27 (39.1%)	
Clinical Stage	I / II	2 (2.9%)	17 (24.6%)	0 (0.0%)	6 (8.7%)	25 (36.2%)	0.098
	III / VI	12 (17.4%)	19 (27.5%)	4 (5.8%)	9 (13.0%)	44 (63.8%)	
Treatment	Surgery	3 (4.3%)	11 (15.9%)	1 (1.4%)	4 (5.8%)	19 (27.5%)	0.807
	Sur. Adj T.^e^	11 (15.9%)	25 (36.2%)	3 (4.3%)	11 (15.9%)	50 (72.5%)	
Metastasis	No	8 (11.6%)	24 (34.8%)	2 (2.9%)	9 (13.0%)	43 (62.3%)	0.790
	Yes	6 (8.7%)	12 (17.4%)	2 (2.9%)	6 (8.7%)	26 (37.7%)	
Loco-regional	No	8 (11.6%)	25 (36.2%)	2 (2.9%)	10 (14.5%)	45 (65.2%)	0.711
Recurrence	Yes	6 (8.7%)	11 (15.9%)	2 (2.9%)	5 (7.2%)	24 (34.8%)	
Lymph node	No	9 (13.0%)	34 (49.3%)	1 (1.4%)	14 (20.3%)	58 (84.0%)	0,011
	Yes	5 (7.2%)	2 (2.9%)	3 (4.3%)	1 (1.4%)	11 (16.0%)	
Death	No	11 (15.9%)	25 (36.2%)	3 (4.3%)	9 (13.0%)	48 (69.6%)	0.556
	Yes	3 (4.3%)	11 (15.9%)	1 (1.4%)	6 (8.7%)	21 (30.4%)	

^a^ +/+ cases CD44+/CD24+; +/− cases CD44+/CD24- and −/− cases CD44-/CD24-; −/− cases CD44-/CD24+. ^b^**PLS:** Primary Lesion Site; ^c^**Major:** Major Salivary Glands **(**Parotid Gland/Submandibular Gland/Sublingual Gland); ^d^**Minor;** Minor Salivary Glands; ^**e**^**Sur. Adj. T.:** Surgery with adjuvant therapy. Statistically significant difference (<0.05) by Fisher’s exact test (two variables) or χ2 (three or more variables) tests.

### Univariate and multivariate analysis of SGMN survival curves

**Figure 2:**
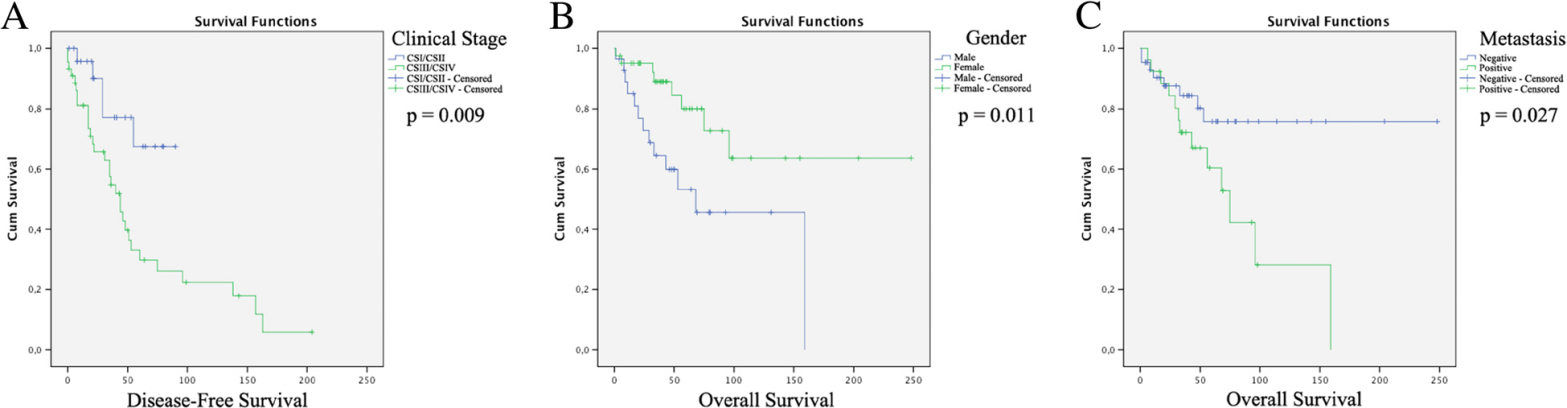
**Univariated analysis. A**) Kaplan-Meier plots of cumulative survival percentage in Disease free survival curve according to SGMN clinical stage in the studied patients. **B**) Kaplan-Meier plots of cumulative survival percentage in Overall survival curve according to patients gender. **C**) Kaplan-Meier plots of cumulative survival percentage in Overall Survival curve according to metastasis event in the studied patients. Statistically significant difference (<0.05).

The Kaplan-Meier analysis establish a significant relationship between gender and OS (p = 0.011) and the present data demonstrate that patients developing metastasis has reduced its OS (p = 0.027). The univariate survival analysis also demonstrated a significant relationship between clinical staging and DFS (p = 0.009). Kaplan–Meier tests also showed that CD44 expression had no statistical significance when correlated to DFS or OS (p = 0.319 and p = 0.447, respectively). CD24 expression, with respect to OS and DFS, had no statistical significance (p = 0.519 and p = 0.081, respectively). The DFS and OS multivariate analysis (Cox proportional hazard regression model) had no statistical significance (p = 0.085 and p = 0.061, respectively). Kaplan–Meier graphics for parameters that had statistical significance are summarized in Figure 2.

## Discussion

The literature shows that clinical staging is the main prognostic factor for SGMN [3,30,31]. Our data are in agreement with this assessment. The present study observed patients in an advanced clinical stage (III/IV) with relation to loco-regional recurrence. In our study, clinical staging also correlated with DFS by Kaplan–Meier tests. The clinical stage was proved to be an independent prognostic factor in the studies of Bell et al. [32] and Lima et al. [33]. Similar, the present study demonstrated that patients in an early clinical stage had a better prognosis.

CD44 is main receptor for hyaluronate. CD44, 80 to 95KDa transmembrane protein has a pivotal role in cell-extracellular matrix interaction [14]. Besides the extracellular interaction with HA, fibronectin, collagen types I and IV, serglycin and osteopontin [16] CD44 forms a complexes with ERM (ezrin-radixin-moesin) to establish a link with actin cytoskeleton [34].

The CD44 receptor is one of the most studied markers for stem cells and is overexpressed in a large number of solid neoplasms. Among the previous CD44 analyses in epithelial tumors, some investigators found relation between this protein and stem cells behavior, suggesting the CD44 as key marker of this cell population [22]. However, recently the role of CD44-expression in solid neoplasms as a tumour marker for stem cells is discussed and controversial.

The relation between CD44 and neoplasms presents the breast cancer as the most well studied neoplasm; an *experimental* assay demonstrates that tumors within CD44-positive cell population possess cancer stem-cell behavior (10). Similar to these results the cancer stem-cell behavior of CD44-positive cells has also demonstrated in head and neck squamous cell carcinomas [22].

Further findings demonstrate that CD44 expression can be related to clinicopathological features and the expression of immunohistochemical markers of prognostic significance. *In an* immunohistochemistry breast invasive ductal carcinoma study Oliveira-Costa and collaborators [35] demonstrated the relation between CD44 expression and HIF1-Alpha status and HER-2 expression influencing the patient prognosis [35]. Choi and collaborators [36] observed an association between CD44 expression and tumour size studding colorectal adenocarcinoma. Notwithstanding, The patients outcome has also been influenced in oral squamous cells carcinoma, the CD44+ subpopulation was a independent factor of a poor prognosis [37]. Nevertheless, the effect of CD44 expression on malignant neoplasms still demonstrates conflicting results, some paper demonstrated that decreased CD44-expression could influence a worse prognosis for the patient [20,21] in endometrioid carcinoma the CD44-expression was observed and no association was found between the protein expression and clinicopathological features suggesting that CD44-expression predictive prognostic factor [38].

Examining salivary glands was demonstrated that CD44v3 and v6 variants are widely expressed by myoepithelial and acinar cells, suggesting that these myoepithelial cells may play a critical role in normal salivary gland renewal and may play a role in the control of growth of salivary gland tumors considered to be derived from modified myoepithelial cells [39]. Franchi and colleagues [40] reported an intense expression of CD44 and CD44v6 in pleomorphic adenomas. This expression was observed in tubular structures, solid areas and areas with myxoid matrix or chondroid matrix production. They also verified that malignant neoplastic cells in carcinoma ex-pleomorphic adenoma cases were positive for CD44. Our data provide an interesting find related between CD44-expression and adenoid cystic carcinoma, was observed an expression of CD44 in myoepithelial component, however, the ductal component presents no expression (Figure 1C). ACC has an indolent nature and predilection to late distant metastasis [41]. ACC shares histopathological features with PMLG. Thus transforming the diagnosis in a challenge to pathologists [42] to decrease the diagnostic difficulty several markers were suggested, an example is the proliferation marker MCM2 suggested by Ghazy and collaborators (2011) [43]. Supported by our Immunohistochemical findings we also suggest that CD44 could be used to differentiate borderline cases. However, further studies with large number of cases are recommended to support the idea. Our data has also provided evidence for a significant association between CD44 expression and SGMN in major salivary glands. This relationship was also suggested by the increased amount of positive staining in major salivary glands tumors when compared with neoplasms from minor salivary glands. A hypothetical explanation for the differential expression of CD44 could be the variation in histological features between the salivary glands and/or embryological development via the epithelium-mesenchyme relationship.

The CD24 receptor is a heavily-glycosylated protein constructed of 27 amino acids attached to the cell membrane by a GPI-anchor [44]. It was described as b-cell marker, and its expression is associated with b-cell development [24,45]. CD24 is related to P-selectin, an adhesion receptor for platelets and activated endothelial cells [24]. The association between CD24 and P-selectin was confirmed due to the interaction of mammary cancer cells and P-selectin in activated platelets [46]. This correlation suggests that CD24 plays an important role in metastatic events in human cancers [23].

CD24 is considered as a new marker for stem cells. This cell surface glycoprotein is grouped together with classical stem cells markers such as CD44, CD133 and CD117. In animal models, the expression of CD24 is observed in submandibular glands [47-49], Naduri and colleagues [49] observed positive staining for CD24 in the submandibular excretory duct. This expression suggests a correlation between CD24 expression and the development of branching epithelium. Our study has shown positive CD24 expression in 9 SGMN cases. On the other hand Ma and colleagues [50] reported that metastatic adenoid cystic carcinoma cell lines lack CD24 expression. Our results, in conjunction with the results of Ma and colleagues [50], indicate that CD24 expression is not well established in SGMN. Our analysis of the CD24-positive cells in SGMN showed an association between CD24 expression, T stage and clinical stage. Cases that expressed CD24 were positively associated TIII/TIV tumors and with cancer stages III / IV. These results may suggest that CD24 correlates with advanced-stage SGMN.

The present study found no correlations between CD24 expression (total, cytoplasmic or membranous) and overall survival. We have also found no correlations between CD24 expression (total, cytoplasmic or membranous) and disease free survival. Oliveira and colleagues [37], studying oral squamous cell carcinoma, have also found no relationship between total CD24 expression and overall survival. However, the CD24-cytoplasmic expression showed a correlation with disease free survival and overall survival. Kristiansen and colleagues [17] highlighted that several studies analyzed only CD24-membranous expression although CD24-cytoplasmatic expression is usually presented as a prognostic factor for human cancer.

Both CD44 and CD24 have been established as stem cell surface markers and have been tested in several groups of tissues and cancers [8-13]. The CD44/CD24 immunophenotypes behavior shows great diversity in human cancers.

In lesions of invasive breast cancer, Mylona and colleagues [51] showed a correlation between the CD44+/CD24- immunophenotype and negative lymph node metastasis and lower staged tumors. However, this result disagrees with the findings of Abraham and coworkers [52] that suggested an association between the CD44+/CD24- immunophenotype and distant metastasis but not with the clinical outcome. In vitro findings suggest that breast cancer cells with lymphatic metastatic ability and expressing the CD44+/CD24- immunophenotype have clonogenic potential and a higher ability to survive and grow in unfavorable environments [53]. In human cases, however, Giatromanolaki and colleagues [54] found that breast cancer patients that present with CD44-/CD24- cells had a worsened overall survival.

Our data suggest that the relationship between CD44 and CD24 may have important role on clinicopathologic features. The immunophenotype CD44+/CD24- was the most prevalent, appearing in 52,2% of salivary glands tumors. Except for the double negative phenotype (CD44-CD24-), all of the immunophenotypes had a prevalent number of cases involving the major salivary glands. This result may suggest a lower activity of stem cells in minor salivary glands neoplasms. This finding could have therapeutic implications. A lower stem cell activity could result in an improved response to therapies aimed at eradicating minor salivary gland tumors. These diverse results reflect the controversial biological characteristics described by Carrillo and coworkers [55] in minor salivary gland neoplasms. Factors that may influence disease free survival rates are histological type, tumor grade and clinical stage [1]. However, clinical stage is the principal prognostic factor for predicting disease outcome [1,3,30,31]. Our findings are in accordance with this literature-derived data.

The present study shows that SGMN with CD44+/CD24+ profile may represents the tumors with most aggressive behavior and worst prognosis. CD44+/CD24+ profiles was associated with tumor size and lymph node metastasis. The majority of the CD44+/CD24+ cases (66,6%) were classified as T3/T4 and the rate of positive lymph node metastasis was higher than the expected (35.7%). Thus, the double positive (CD44+/CD24+) profile may be correlated with the more advanced tumors and rapid disease development. This aggressive cell behavior was also demonstrated in CD44+/CD24+ pancreatic cancer cells. Injection of CD44+/CD24+ cells into an animal model resulted in tumors a mere 3 weeks after injection. The same results were not achieved with CD44-/CD24- pancreatic cancer cells. These data demonstrate the tumorigenic potential of pancreatic cancer cells bearing the CD44+/CD24+ immunophenotype [12]. This phenomenon was also observed in the study conducted by Huang and coworkers [56], where CD44+/CD24+ pancreatic cancer cells showed an exacerbated tumorigenic potential. Because stem cells from salivary gland tissue and pancreatic tissue have similar capacities for proliferation and differentiation, due to their similar aggressive capacity, we can assume that the cancer stem cell from both tissues also has similar abilities [57].

## Conclusion

In summary, our investigation confirms that the clinical stage, in accordance with the literature, is the main prognostic factor for SGMN. Additionally, we have presented some evidence that the analysis of isolated CD44 and CD24 immunoexpression or the CD44/CD24 immunophenotypes could give a prognostic informations associated to clinicopathologic features Salivary Gland Malignant Neoplasms.

## Competing interests

The authors declare that they have no competing interests.

## Authors’ contributions

DFS participated in the sequence alignment, performed the statistical analysis and drafted the manuscript. GGS participated in the sequence alignment. JPOC participated in the design of the study and performed the statistical analysis. RCFI participated in the design of the study. LRO conceived of the study, and participated in its design. SVL conceived the study and participated in its design. ARS conceived of the study, and participated in its design of the study and coordination and helped to draft the manuscript. All authors read and approved the final manuscript.

## References

[B1] Lopes MA Santos GC Kowalski LP Multivariate survival analysis of 128 cases of oral cavity minor salivary gland carcinomas Head Neck 1998 20 8 699 706 10.1002/(SICI)1097-0347(199812)20:8<699::AID-HED7>3.0.CO;2-P 9790291

[B2] Speight PM Barrett AW Prognostic factors in malignant tumours of the salivary glands Br J Oral Maxillofac Surg 2009 47 8 587 593 10.1016/j.bjoms.2009.03.017 19409681

[B3] Hocwald E Korkmaz H Yoo GH Adsay V Shibuya TY Abrams J Jacobs JR Prognostic factors in major salivary gland cancer Laryngoscope 2001 111 8 1434 1439 10.1097/00005537-200108000-00021 11568581

[B4] Phillips TM McBride WH Pajonk F The response of CD24(−/low)/CD44+ breast cancer-initiating cells to radiation J Natl Cancer Inst 2006 98 24 1777 1785 10.1093/jnci/djj495 17179479

[B5] Cariati M Evaluating the link between stem cells and breast cancer Expert Rev Anticancer Ther 2008 8 8 1313 1322 10.1586/14737140.8.8.1313 18699767

[B6] Oliveira L Jeffrey S Ribeiro-Silva A Stem cells in human breast cancer Histol Histopathol 2010 25 3 371 385 20054808 10.14670/HH-25.371

[B7] Umezawa A Gorham JD Dueling models in head and neck tumor formation Lab Invest 2010 90 11 1546 1548 10.1038/labinvest.2010.165 21030945

[B8] Singh SK Hawkins C Clarke ID Squire JA Bayani J Hide T Henkelman RM Cusimano MD Dirks PB Identification of human brain tumour initiating cells Nature 2004 432 7015 396 401 10.1038/nature03128 15549107

[B9] Ricci-Vitiani L Lombardi DG Pilozzi E Biffoni M Todaro M Peschle C De Maria R Identification and expansion of human colon-cancer-initiating cells Nature 2007 445 7123 111 115 10.1038/nature05384 17122771

[B10] Al-Hajj M Wicha MS Benito-Hernandez A Morrison SJ Clarke MF Prospective identification of tumorigenic breast cancer cells Proc Natl Acad Sci USA 2003 100 7 3983 3988 10.1073/pnas.0530291100 12629218 PMC153034

[B11] Fang D Nguyen TK Leishear K Finko R Kulp AN Hotz S Van Belle PA Xu X Elder DE Herlyn M A tumorigenic subpopulation with stem cell properties in melanomas Cancer Res 2005 65 20 9328 9337 10.1158/0008-5472.CAN-05-1343 16230395

[B12] Li C Heidt DG Dalerba P Burant CF Zhang L Adsay V Wicha M Clarke MF Simeone DM Identification of pancreatic cancer stem cells Cancer Res 2007 67 3 1030 1037 10.1158/0008-5472.CAN-06-2030 17283135

[B13] Patrawala L Calhoun T Schneider-Broussard R Li H Bhatia B Tang S Reilly JG Chandra D Zhou J Claypool K Highly purified CD44+ prostate cancer cells from xenograft human tumors are enriched in tumorigenic and metastatic progenitor cells Oncogene 2006 25 12 1696 1708 10.1038/sj.onc.1209327 16449977

[B14] Shtivelman E Bishop JM Expression of CD44 is repressed in neuroblastoma cells Mol Cell Biol 1991 11 11 5446 5453 1922057 10.1128/mcb.11.11.5446-5453.1991 PMC361913

[B15] De Marzo AM Bradshaw C Sauvageot J Epstein JI Miller GJ CD44 and CD44v6 downregulation in clinical prostatic carcinoma: relation to Gleason grade and cytoarchitecture Prostate 1998 34 3 162 168 10.1002/(SICI)1097-0045(19980215)34:3<162::AID-PROS2>3.0.CO;2-K 9492843

[B16] Assimakopoulos D Kolettas E Patrikakos G Evangelou A The role of CD44 in the development and prognosis of head and neck squamous cell carcinomas Histol Histopathol 2002 17 4 1269 1281 12371152 10.14670/HH-17.1269

[B17] Kristiansen G Machado E Bretz N Rupp C Winzer KJ König AK Moldenhauer G Marmé F Costa J Altevogt P Molecular and clinical dissection of CD24 antibody specificity by a comprehensive comparative analysis Lab Invest 2010 90 7 1102 1116 10.1038/labinvest.2010.70 20351695

[B18] Heider KH Dämmrich J Skroch-Angel P Müller-Hermelink HK Vollmers HP Herrlich P Ponta H Differential expression of CD44 splice variants in intestinal- and diffuse-type human gastric carcinomas and normal gastric mucosa Cancer Res 1993 53 18 4197 4203 7689929

[B19] Wielenga VJ Heider KH Offerhaus GJ Adolf GR van den Berg FM Ponta H Herrlich P Pals ST Expression of CD44 variant proteins in human colorectal cancer is related to tumor progression Cancer Res 1993 53 20 4754 4756 7691404

[B20] Sato S Miyauchi M Takekoshi T Zhao M Kudo Y Ogawa I Kitagawa S Fujita M Takata T Reduced expression of CD44 variant 9 is related to lymph node metastasis and poor survival in squamous cell carcinoma of tongue Oral Oncol 2000 36 6 545 549 10.1016/S1368-8375(00)00049-X 11036249

[B21] Kosunen A Pirinen R Ropponen K Pukkila M Kellokoski J Virtaniemi J Sironen R Juhola M Kumpulainen E Johansson R Nuutinen J Kosma VM CD44 expression and its relationship with MMP-9, clinicopathological factors and survival in oral squamous cell carcinoma Oral Oncol 2007 43 1 51 59 10.1016/j.oraloncology.2006.01.003 16798062

[B22] Prince ME Sivanandan R Kaczorowski A Wolf GT Kaplan MJ Dalerba P Weissman IL Clarke MF Ailles LE Identification of a subpopulation of cells with cancer stem cell properties in head and neck squamous cell carcinoma Proc Natl Acad Sci USA 2007 104 3 973 978 10.1073/pnas.0610117104 17210912 PMC1783424

[B23] Weichert W Denkert C Burkhardt M Gansukh T Bellach J Altevogt P Dietel M Kristiansen G Cytoplasmic CD24 expression in colorectal cancer independently correlates with shortened patient survival Clin Cancer Res 2005 11 18 6574 6581 10.1158/1078-0432.CCR-05-0606 16166435

[B24] Lim SC CD24 and human carcinoma: tumor biological aspects Biomed Pharmacother 2005 59 Suppl 2 S351 S354 16507407 10.1016/s0753-3322(05)80076-9

[B25] Kristiansen G Pilarsky C Pervan J Stürzebecher B Stephan C Jung K Loening S Rosenthal A Dietel M CD24 expression is a significant predictor of PSA relapse and poor prognosis in low grade or organ confined prostate cancer Prostate 2004 58 2 183 192 10.1002/pros.10324 14716744

[B26] Barnes L Everson J Reichart P Sidransky D Kleihues P, Sobin LH Pathology and Genetics or Head and Neck Tumours “World Health Organization Classification of Tumours 2005 Lyon: IARC Press 209 281

[B27] Wittekind C Greene FL Hutter RVP Klimpfinger M Sobin LH Wittekind C, Greene FL, Hutter RVP, Klimpfinger M, Sobin LH Illustrated Guide to the TNM/pTNM Classification of Malignant Tumours”TNM Atlas” 2005 New York: Springer Verlag 61 65

[B28] Honeth G Bendahl PO Ringnér M Saal LH Gruvberger-Saal SK Lövgren K Grabau D Fernö M Borg A Hegardt C The CD44+/CD24- phenotype is enriched in basal-like breast tumors Breast Cancer Res 2008 10 3 R53 10.1186/bcr2108 18559090 PMC2481503

[B29] Kristiansen G Winzer KJ Mayordomo E Bellach J Schlüns K Denkert C Dahl E Pilarsky C Altevogt P Guski H CD24 expression is a new prognostic marker in breast cancer Clin Cancer Res 2003 9 13 4906 4913 14581365

[B30] Bhattacharyya N Fried MP Nodal metastasis in major salivary gland cancer: predictive factors and effects on survival Arch Otolaryngol Head Neck Surg 2002 128 8 904 908 12162768 10.1001/archotol.128.8.904

[B31] Guzzo M Locati LD Prott FJ Gatta G McGurk M Licitra L Major and minor salivary gland tumors Crit Rev Oncol Hematol 2010 74 2 134 148 10.1016/j.critrevonc.2009.10.004 19939701

[B32] Bell RB Dierks EJ Homer L Potter BE Management and outcome of patients with malignant salivary gland tumors J Oral Maxillofac Surg 2005 63 7 917 928 10.1016/j.joms.2005.03.006 16003616

[B33] Lima RA Tavares MR Dias FL Kligerman J Nascimento MF Barbosa MM Cernea CR Soares JR Santos IC Salviano S Clinical prognostic factors in malignant parotid gland tumors Otolaryngol Head Neck Surg 2005 133 5 702 708 10.1016/j.otohns.2005.08.001 16274796

[B34] Rudzki Z Jothy S CD44 and the adhesion of neoplastic cells Mol Pathol 1997 50 2 57 71 10.1136/mp.50.2.57 9231152 PMC379585

[B35] Oliveira-Costa JP Zanetti JS Silveira GG Soave DF Oliveira LR Zorgetto VA Soares FA Zucoloto S Ribeiro-Silva A Differential expression of HIF-1alpha in CD44 + CD24-/low breast ductal carcinomas Diagn Pathol 2011 6 73 10.1186/1746-1596-6-73 21824412 PMC3170242

[B36] Choi D Lee HW Hur KY Kim JJ Park GS Jang SH Song YS Jang KS Paik SS Cancer stem cell markers CD133 and CD24 correlate with invasiveness and differentiation in colorectal adenocarcinoma World J Gastroenterol 2009 15 18 2258 2264 10.3748/wjg.15.2258 19437567 PMC2682242

[B37] Oliveira LR Oliveira-Costa JP Araujo IM Soave DF Zanetti JS Soares FA Zucoloto S Ribeiro-Silva A Cancer stem cell immunophenotypes in oral squamous cell carcinoma J Oral Pathol Med 2010 40 135 21073537 10.1111/j.1600-0714.2010.00967.x

[B38] Gun BD Bahadir B Bektas S Barut F Yurdakan G Kandemir NO Ozdamar SO Clinicopathological significance of fascin and CD44v6 expression in endometrioid carcinoma Diagn Pathol 2012 7 80 10.1186/1746-1596-7-80 22784357 PMC3407727

[B39] Fonseca F Moura Nunes JF Soares J Expression of CD44 isoforms in normal salivary gland tissue: an immunohistochemical and ultrastructural study Histochem Cell Biol 2000 114 6 483 488 11201610 10.1007/s004180000220

[B40] Franchi A Moroni M Paglierani M Santucci M Expression of CD44 standard and variant isoforms in parotid gland and parotid gland tumours J Oral Pathol Med 2001 30 9 564 568 10.1034/j.1600-0714.2001.300910.x 11555161

[B41] Zhou Q Chang H Zhang H Han Y Liu H Increased numbers of P63-positive/CD117-positive cells in advanced adenoid cystic carcinoma give a poorer prognosis Diagn Pathol 2012 7 119 10.1186/1746-1596-7-119 22963430 PMC3487809

[B42] Speight PM Barrett AW Salivary gland tumours Oral Dis 2002 8 5 229 240 10.1034/j.1601-0825.2002.02870.x 12363107

[B43] Ghazy SE Helmy IM Baghdadi HM Maspin and MCM2 immunoprofiling in salivary gland carcinomas Diagn Pathol 2011 6 89 10.1186/1746-1596-6-89 21943228 PMC3191357

[B44] Sagiv E Starr A Rozovski U Khosravi R Altevogt P Wang T Arber N Targeting CD24 for treatment of colorectal and pancreatic cancer by monoclonal antibodies or small interfering RNA Cancer Res 2008 68 8 2803 2812 10.1158/0008-5472.CAN-07-6463 18413748

[B45] Fogel M Friederichs J Zeller Y Husar M Smirnov A Roitman L Altevogt P Sthoeger ZM CD24 is a marker for human breast carcinoma Cancer Lett 1999 143 1 87 94 10.1016/S0304-3835(99)00195-0 10465342

[B46] Aigner S Ramos CL Hafezi-Moghadam A Lawrence MB Friederichs J Altevogt P Ley K CD24 mediates rolling of breast carcinoma cells on P-selectin FASEB J 1998 12 12 1241 1251 9737727 10.1096/fasebj.12.12.1241

[B47] Shirasawa T Akashi T Sakamoto K Takahashi H Maruyama N Hirokawa K Gene expression of CD24 core peptide molecule in developing brain and developing non-neural tissues Dev Dyn 1993 198 1 1 13 10.1002/aja.1001980102 8292828

[B48] Akashi T Shirasawa T Hirokawa K Gene expression of CD24 core polypeptide molecule in normal rat tissues and human tumor cell lines Virchows Arch 1994 425 4 399 406 7820302 10.1007/BF00189578

[B49] Nanduri LS Maimets M Pringle SA van der Zwaag M van Os RP Coppes RP Regeneration of irradiated salivary glands with stem cell marker expressing cells Radiother Oncol 2011 99 3 367 372 10.1016/j.radonc.2011.05.085 21719134

[B50] Ma YQ Geng JG Obligatory requirement of sulfation for P-selectin binding to human salivary gland carcinoma Acc-M cells and breast carcinoma ZR-75-30 cells J Immunol 2002 168 4 1690 1696 11823498 10.4049/jimmunol.168.4.1690

[B51] Mylona E Giannopoulou I Fasomytakis E Nomikos A Magkou C Bakarakos P Nakopoulou L The clinicopathologic and prognostic significance of CD44+/CD24(−/low) and CD44-/CD24+ tumor cells in invasive breast carcinomas Hum Pathol 2008 39 7 1096 1102 10.1016/j.humpath.2007.12.003 18495204

[B52] Abraham BK Fritz P McClellan M Hauptvogel P Athelogou M Brauch H Prevalence of CD44+/CD24-/low cells in breast cancer may not be associated with clinical outcome but may favor distant metastasis Clin Cancer Res 2005 11 3 1154 1159 15709183

[B53] Pandit TS Kennette W Mackenzie L Zhang G Al-Katib W Andrews J Vantyghem SA Ormond DG Allan AL Rodenhiser DI Lymphatic metastasis of breast cancer cells is associated with differential gene expression profiles that predict cancer stem cell-like properties and the ability to survive, establish and grow in a foreign environment Int J Oncol 2009 35 2 297 308 19578743

[B54] Giatromanolaki A Sivridis E Fiska A Koukourakis MI The CD44+/CD24- phenotype relates to 'triple-negative' state and unfavorable prognosis in breast cancer patients Med Oncol 2011 28 3 745 752 10.1007/s12032-010-9530-3 20405247

[B55] Carrillo JF Maldonado F Carrillo LC Ramirez-Ortega MC Pizano JG Melo C Chanona JG Luna-Ortiz K Ocaña LF Prognostic factors in patients with minor salivary gland carcinoma of the oral cavity and oropharynx Head Neck 2011 33 10 1406 1412 10.1002/hed.21641 21928413

[B56] Huang P Wang CY Gou SM Wu HS Liu T Xiong JX Isolation and biological analysis of tumor stem cells from pancreatic adenocarcinoma World J Gastroenterol 2008 14 24 3903 3907 10.3748/wjg.14.3903 18609717 PMC2721450

[B57] Gorjup E Danner S Rotter N Habermann J Brassat U Brummendorf TH Wien S Meyerhans A Wollenberg B Kruse C Glandular tissue from human pancreas and salivary gland yields similar stem cell populations Eur J Cell Biol 2009 88 7 409 421 10.1016/j.ejcb.2009.02.187 19410331

